# A novel bi-level programming model for structure sustainability optimization of passenger transport corridor

**DOI:** 10.1371/journal.pone.0266013

**Published:** 2022-07-08

**Authors:** Xu Wang, Jingni Song, Qunqi Wu, Yifeng Bao, Yiping Wang

**Affiliations:** 1 School of Economics and Management, Chang’an University, Xi’an, China; 2 College of Transportation Engineering, Chang’an University, Xi’an, China; 3 School of Electronics and Control Engineering, Chang’an University, Xi’an, China; University of Defence in Belgrade, SERBIA

## Abstract

The transportation industry has entered a new stage from quantity expanding to structure optimization, quality and efficiency improvement, and from respective governance to integrative development. This indicates that the traditional corridor mode allocation dominated by quantity equilibrium can no longer meet the requirements of the new stage. In this paper, we propose a multi-entity programming model based on the economic equilibrium between supply and demand. It not only ensures the economic equilibrium in the market, but also maximizes the social benefits of the whole system, thereby realizing the sustainable development of the transportation system. Also, the Globalsearch algorithm and intlinprog algorithm are designed to solve the problem. The actual case of Beijing-Shanghai corridor shows that the model and algorithms are effective, providing decision support for the optimal allocation of regional transport network resources.

## Introduction

In the past few years, countries around the world have taken the construction of high-speed railway as one of the key strategies for national or regional development. Japan is one the first countries to plan, construct and operate such railways. In 1964, the rail between Tokyo and Osaka began to operate, which started the era of high-speed rail construction and operation. Subsequently, France, Germany, Canada, Chinese mainland and Taiwan ushered in a new era of building high-speed rail one after another. Among them, China plays the leading role in terms of construction speed and scale. Recently, China State Railway Group Co., Ltd. released the Outline of Powerful Nation Railway Advance Planning in the New Era [1], which has planned the construction of an HSR network about 70,000 km in 2035. However, empirical analysis shows that with the rapid development of high-speed rail, the phenomenon of “involuntary high-speed railway travel” is relatively common in China. It means that people of modest means may have to pay a high fare to take high-speed trains. The extent of this phenomenon varies in different cities, but the possibility is around 50% on average [2]. In addition, it is found that high-speed rail is a strong competitor of civil aviation, which has seen fewer flights as well as lower market share and profit [3].

We can see that with the rapid development of different transport modes, problems such as resource preemption, redundant construction, and waste of capacity are increasingly prominent [4]. Practice shows that the structural surplus of transportation in China has become a severe problem so far. In addition to the management system and mechanism [5], the traditional planning method is also a factor that restricts its development [6]. It is also one of the problems that planners and practitioners have been puzzled and interested in for a long time. Therefore, this article focuses on the capacity configuration of different modes in a regional passenger transportation corridor.

At present, the allocation of corridor modes is mainly based on themselves, making their own construction plans and realizing expansion. This is necessary and feasible in the period of traffic shortage. However, when different modes develop to a certain scale, the difference and individualization of transportation demand are becoming more and more obvious, which inevitably requires the in-depth integration of various transportation modes. Practice has shown that traditional methods of configuration have resulted in disharmony and waste of resources, and it is difficult to achieve the basic objective of a comprehensive transportation system in the new stage. Therefore, it is urgently important to explore the theory and method of optimal allocation of corridor modes adapted to the characteristics of the new stage. From the perspective of overall planning, this paper attempts to construct a bi-level programming model of regional passenger corridor optimization configuration considering multi-entity and multi-criteria based on the theory of consumer surplus. Also, algorithms are designed to solve the problem, so as to provide guidance for related management departments.

As is known, the passenger transport system in comprehensive transportation corridor includes two basic levels: infrastructure and transport activities. The former mainly depends on the government, while the latter is completely market-oriented. Despite the difference in their leading drivers, only a combination of the two levels can form the transport service capacity. Therefore, when promoting infrastructure planning, the government must make full use of the operating rules of market economy, and make it clear that demand is primary and dominant. In doing so, it can meet the social demands to the maximum extent. It provides services according to the real situation, choosing the most appropriate way. But the mode of transport service is determined by the economic equilibrium between the supply and demand entities. Economic equilibrium refers to the internal economic state in which the two entities make a deal on the basis of satisfying their own interests. They have their own utility or price bottom line constraints. When the displacement demand breaks the bottom line and the price is lower than the cost, the transaction will not be possible. In this case, there is no economic equilibrium between supply and demand. Therefore, in the process of optimizing the layout of regional transport corridor, there are three different interest entities, namely, corridor planner (government), transport supplier (transport enterprise) and demand entity (passenger), which are interrelated with each other. This study aims at tackling this issue by focusing on the overall planning, and proposes a new configuration method that meets the interests of all entities, including the government, supply entity and demand entity. In fact, this configuration decision process is a system optimization problem with a two-level hierarchical structure, and its essence is a bi-level programming model. We believe that the key to future transport mode allocation is to judge which one can meet the demand to the maximum extent and make the best use of social resources on the basis of the changing trend of corridor demand, the evolution of corridor structure and the constraints of social resources [7]. This is also the key difference between this article and traditional research, making it more suitable for the characteristics of market economy operation.

To the best of our knowledge, there is almost no relevant research about this problem, except the research of Song (2019) [8]. She is the first to apply the idea of economic equilibrium to the optimization of regional passenger transport corridor, changing the traditional corridor mode optimization research status dominated by quantity equilibrium. It is a new attempt and breakthrough. Our contribution in this paper is fourfold. First, we formulate the corridor structure optimization problem from the perspective of three different stakeholders. This problem is oriented to the planning and decision-making process. Song only tentatively proposed to analyze the corridor optimization problem with the idea of economic equilibrium between supply and demand, and only considered the economic interaction between supply and demand entities. In fact, there are three different interest entities in this process. The final plan depends on the government, so its interests should also be fully considered. Second, we also comprehensively consider such factors as the transformation of existing transport networks, the layout of new networks and the technical level of lines into account, which have not been considered by the existing research. Third, we reckon this problem as a two-level programming model, and design an appropriate algorithm to solve it. Fourth, we test our model on one case study based on the Beijing-Shanghai transport corridor, so as to provide decision support for industry management departments.

This research has important academic values in terms of improving passenger transportation corridor structure optimization in region-specific comprehensive transport that conforms to a market economy mechanism. This concept can be extended from single corridor planning to point-to-point and door-to-door transportation supply structure planning, and to comprehensive transport network planning and urban transportation planning without loss of generality. It has important guiding significance for government departments to make better infrastructure planning and operating companies to provide more efficient transportation services.

The remainder of this paper is organized as follows. Section 2 is the literature review. In Section 3, we describe the problem to be studied and put forward the model hypothesis. Section 4 constructs a bi-level programming model framework and designs algorithms to solve it. In Section 5, we analyze the case of Beijing-Shanghai corridor for managerial insights. Finally, Section 6 presents concluding remarks.

## Literature review

According to the outline of this paper, the literature review is divided into three parts.

### Cross-regional transport planning

Cross-regional trips are those from one regional jurisdiction to another which are served by different modes. So far, there is little literature available on this topic [9], and studies on cross-regional transportation planning is even rarer. Just as Ryan put it, regional transport is the forgotten step child of transportation behavioural research [10].

The traditional transportation planning theory has become increasingly mature, especially urban transport planning, which originated from the research of several large cities such as the Detroit metropolitan area and the Chicago metropolitan area in the 1950s. After more than half a century of development, it gradually formed a theoretical framework based on the four-stage planning method. Due to the problems that occured later, the larger-scale regional transportation planning theory research lagged behind the urban transportation planning, and even earlier regional transportation planning basically copied the urban transportation planning theories and methods directly. Based on the standards specified by different modes, it compared the current situation with future traffic demand, and took the difference between them as the basis for traffic planning [6]. There is a big difference between regional and urban traffic demands, and the networks have their own characteristics in terms of infrastructure and transport products. Therefore, it remains to be proved whether urban traffic planning theories and methods are fully applicable to regional traffic planning [6].

Some researchers have focused on long distance trips, like Barma et al.(2019) [11], Roy et al.(2019) [12], Sfm et al.(2020) [13] and Rashedi et al. (2017) [9]. However, these trips do not necessarily represent intercity transport planning. There are also some scholars who have studied the planning of different transport modes in the cross-regions [14–16]. But the addition of all the planning is not the overall comprehensive transport planning. In addition, this kind of planning is passively satisfying, which only meets the demand from the perspective of quantity [8]. As an activity of market economy, the internal economic equilibrium should be the fundamental basis for the formation of transactions.

The type of intercity transport service, in general, should be determined mainly by the demand and the services, although other considerations, such as budget constraint, can may play an important role in this process [7]. To accurately estimate the passenger flow of different modes, it is necessary to develop a model that includes policy-sensitive variables and can capture the differences in the internal preference and sensitivity of individuals to the change of service level.

### Selection of transport modes

Several transport modes are available in the literature, including ordinary rail, high-speed rail, air and highway. These modes differ in several ways, such as the capital cost, operating cost, transport capacity, and travel speed. Why do people choose Mode A instead of B or C? This has been been a research question in economics and social sciences [17], which can be traced back to the 1950s [6]. Subsequently, a large number of scholars conducted extensive research on it and have gradually developed two models: aggregate model and disaggregate model. The latter is based on the utility maximization hypothesis, which assumes that an individual’s choice is a reflection of his underlying preferences for each of the available alternatives and that the individual selects the mode with the highest preference or utility [18]. This model has become an important means of transport demand analysis because of its high efficiency, low cost and convenient modeling. The most widely used disaggregate model can be divided into two categories. One is based on the multinomial logit model and its improved forms [9, 19, 20], and the other is Probit model [21, 22]. With the development of discrete choice modeling techniques and simulation algorithm, the Bayesian parameter estimation method has seen rapid development and wide application in the field of transport studies [23–25].

In addition, Chander et al. proposed a decision-making method using an IVPFS-based similarity measure for choosing the right alternative under uncertain emergency decision making [26]. On this basis, they presented a multi-attribute decision-making approach based on interval-valued pythagorean fuzzy set and differential evolutionary algorithm [27]. Also, Sahu et al. proposed a PFS-based approach which uses the hybridized distance measure for choosing a suitable and appropriate career for a student [28]. These research methods also have important reference significance for the choice of transportation mode.

These models provide strong theoretical and methodological support for the study of passengers’ travel mode choice. However, they have two obvious limitations in guiding the corridor mode planning. First, these models are based on the utility maximization theory, which studies the travel choice on the basis of existing modes. The best choice from limited supply is not always the most satisfactory one. Second, travel utility includes absolute utility and relative utility. According to utility theory, utility maximization is the core of decision criteria. In the labor market, consumers realize the conversion from money to utility by purchasing goods, which is a direct conversion. However, in the field of transportation, consumers can not purchase by experiencing it due to the particularity of displacement. It can be achieved by satisfying the demand entity after finishing the displacement, which is an indirect conversion. Generally, the disaggregate model is a utility value that comprehensively calculates the safety, comfort and economy brought by a travel mode. But it only calculates the relative utility of the mode, not the absolute utility that the realization of displacement really brings to the demand entity. Therefore, this study proposes to explore the mode configuration of passenger transport corridor based on the consumer surplus theory.

### Summary

According to the literature review, we find the following results:

- Quite a lot of research focuses on urban transportation mode planning, and gradually forms a theoretical framework based on the four-stage planning method, while relatively few studies concentrate on regional corridor mode planning, which mainly focus on single-mode planning research.
- It has been recognized that the choice of transport modes depends mainly on the demand pattern, but it is not appropriate to use it to guide cross-regional mode planning. In our view, corridor mode planning is affected by multiple factors, such as the government, the demand and transit services, but the economic equilibrium between supply and demand is the core.
- The existing literature is more based on the utility maximization theory to analyze the impact of demand, which has obvious limitations (as shown above). We believe that the consumer surplus theory has more advantages in analyzing the mechanism of demand selection.

## Problem description

The research problem is to optimize the transport capacity structure of regional passenger transport corridor by using economic theories and system analysis method. In addition to the internal economic laws among entities in the transport market, it also needs to consider the requirements of the planners, or, government departments, and the relationship between the transformation of existing transport networks and the layout of new networks. It is a complicated task, because there are three different interest entities in the process: corridor planner (government), network user (transportation enterprise) and user (passenger), and their goals are different. How to seek a balance between them is the key to be solved in this paper. However, the transformation and new construction of networks are also affected by various factors, such as capacity constraints, capital constraints, environmental energy consumption constraints, land resources constraints and so on. In addition, the transportation demand is unbalanced in time and space, so it is necessary to simplify its characteristics in this study.

In order to clarify the problem, we assume that the passenger transport corridor in an area is composed of A pairs of O-D. O is the starting point, and D is the destination, and they are in different areas. There are *n* categories of travel demand entities in the corridor, and *m* modes of transportation are considered. The total demand is *N*, and *a* ∈ *A*, travel demand entity *i* ∈ *I*, transport mode *j* ∈ *J*. The planners seek to maximize the social benefits of the entire system in the corridor under certain constraints such as construction cost investment, environmental carrying capacity and energy consumption. As for the demand entities, under actual supply conditions, they always want to choose the mode *j* that can realize the largest consumer surplus. But when its supply is insufficient, they can only choose mode *k* (*k*≠*j*) that has a larger consumer surplus than others. If the travel surplus is negative, the demand entity will give up traveling. For the supply entities, if the demand intensity of a certain mode can not reach the break-even point in the planning period, the demand will not be met temporarily. Therefore, the organic unity of the interest functions of the demand and supply entities is the key to optimizing the supply structure of passenger transport corridor mode. For more about the economic equilibrium mechanism between supply and demand, please refer to Song (2019) [8]. In the process of optimization, the planners comprehensively consider the impacts of construction cost, environment and energy consumption on the basis of economic equilibrium. And then they decide whether to build or transform a certain mode.

To simplify the problem, the assumptions of the model are given below:

1. We only talk about the capacity allocation problem of different transport modes in parallel connection, and combined transportation will be dismissed in this paper.
2. The travel demand entity tends to choose the service mode and transport mode with the largest travel surplus, which is called the hypothesis of rational man.
3. It is assumed that in the transportation connection between two nodes of regional passenger transport corridor, a certain line of a transport mode is transformed to the higher level by default [29]. And the reconstructed mileage is consistent with the original one.

## Bi-objective framework

In this section, we formalize the hybrid network design problem and propose a method based on heuristic algorithm to solve the mathematical procedures in Section 4.2. The notations are shown in Table 1.

**Table 1:** Summary of notations.

Notation	Description
*A*	Set of O-D pairs
*P*_*aj*_	The price of the *j*th mode in the *a*th O-D pair
$\delta_{a}^{ij}$	Ratio for the *i*th demand entity to select the jth mode in the *a*th O-D pair
* $X_{a}^{jr}$ *	0–1 variable, which is 1 if a new mode *j* with grade *r* is constructed between the *a*th O-D pair; otherwise, it is 0.
* $Y_{a}^{jr}$ *	0–1 variable, 1 if the line grade of the mode *j* is raised by one level; otherwise, it is 0.
*n*	Number of categories for demand entities
*m*	Number of transportation modes
$L_{doc}{}_{a}^{jr}$	Line length of the mode *j* with grade *r* between the *a*th O-D pair
$L_{a}^{jr}$	Planned line mileage of the mode *j* with grade *r* between the *a*th O-D pair
*L*_0_	Existing line mileage
$h_{a}^{jr}$	Unit cost of constructing new mode *j* with grade *r* between the *a*th O-D pair
$g_{a}^{jr}$	Unit cost of transforming the line of the mode *j* into grade *r* between the *a*th O-D pair
$q_{a}^{jr}$	Passenger flow of the mode *j* with grade *r* between the *a*th O-D pair
*B*	Total investment budget for network reconstruction and expansion in the corridor
$DC_{doc}{}_{a}^{jr}$	Designed transport capacity of the mode *j* with grade *r* between the *a*th O-D pair
$DC_{a}^{jr}$	Designed transport capacity of all planned routes of the mode *j* with grade *r* between the *a*th O-D pair
*DC*_0_	Designed transport capacity of existing lines
*α*	Transport capacity reserve factor, generally 0.85
$\lambda_{a}^{jf}(v_{a}^{j})$	Emission function of the pollutant *f* when the mode *j* between the *a*th O-D pair runs, and f = 1, 2 and 3, represent CO, NO_x_ and HC, respectively
*χ*_*f*_	Unit cost for the treatment of the *f*th pollutant
$E_{a}^{j}$	Unit energy consumption of the *j*th mode
$e_{a}^{j}$	Unit energy consumption of the *j*th mode(converted to joules)
*φ*	Economic conversion coefficient of energy consumption
$p_{a}^{j}$	Fare rate of the *j*th mode
$F_{a}^{j}$	Fixed cost of mode *j* in the *a*th O-D pair
$F_{0}{}_{a}^{j}$	Current fixed cost of mode *j* in the *a*th O-D pair
$q_{a}^{j}$	Number of passengers demanded in mode *j* in the *a*th O-D pair
$AV_{a}^{j}$	Unit variable cost of the *j*th mode in the *a*th O-D pair
$R_{a}^{j}$	Equilibrium rate of return for the *j*th mode in the *a*th O-D pair
$V_{a}^{i}$	Travel value of the *i*th travel demand entity in the *a*th O-D pair
* $C_{a}^{ij}$ *	Travel cost for the *i*th travel demand entity to select the *j*th mode in the *a*th O-D pair
* $\beta_{a}^{i}$ *	Time value cost coefficient for the *i*th travel demand entity in the *a*th O-D pair
$\gamma_{a}^{i}$	Other psychological cost coefficient for the *i*th travel demand entity in the *a*th O-D pair
* $N_{a}^{i}$ *	Number of the *i*th travel demand entity in the *a*th O-D pair
$t_{a}^{i}$	Travel time of the *j*th mode in the *a*th O–D pair

### Model formulation

Model 1 (upper model): In the investment scope and under the constrains of environmental carrying capacity and energy consumption, the system has the greatest social benefit. The model is:

$$\text{min}Z=\sum_{a\in A}\sum_{j=1}^{m}\sum_{r}\left(L_{doc}{}_{a}^{jr}h_{a}^{jr}X_{a}^{jr}+L_{doc}{}_{a}^{jr}g_{a}^{jr}Y_{a}^{jr}\right)+\sum_{f}\sum_{a\in A}\sum_{j=1}^{m}\sum_{r}\chi_{f}\lambda_{a}^{jf}(v_{a}^{j})q_{a}^{j\text{r}}L_{a}^{jr}(X,L_{0})+\sum_{a\in A}\sum_{j=1}^{m}\sum_{r}\phi_{a}^{j}E_{a}^{j}q_{a}^{jr}L_{a}^{jr}(X,L_{0})$$ (1)

s.t.∑a∈A∑j=1m∑r(φLajrhajr+μLajrgajr)≤B(a)∑a∈A∑j=1m∑f=1∑rλajfqajLajr≤Fmax(b)∑a∈A∑j=1m∑reajqajLajr≤Hemax(c)α·∑rDCajrXajr+DCajrYajr≥qaj,∀a∈A(d)∑j=1mqaj(Xajr,Yajr,Paj)≥Na,∀a∈A(e)∑a∈A∑j=1m(LajrXajr+La0j)≤Lj(f)Xajr∈0,1,Yajr∈0,1,∀a∈Aqaj=∑i=1nNaiδaij,∀a∈ALajr(X,L0)=LdocajrXajr+L0,∀a∈ADCajr=DC0+DCdocajrXajr+ΔDCdocajrYajr,∀a∈A(g)

Constraint (a) ensures that the cost of the new construction and transformation of different modes is less than the total investment. Constraint (b), from the perspective of sustainable development, indicates that the total pollution of a passenger transport corridor cannot exceed the limit of environmental capacity, and *F*_max_ is the limit of pollutant discharge. Constraint (c) ensures that the total energy consumption is less than the limit value *H*_emax_. Constraint (d) holds that the transport capacity of the *j*th mode should not be less than the demand. Constraint (e) demonstrates that the overall transport capacity of a corridor must be no less than the total demand. Constraint (f) makes sure that the new constructed, transformed and original mileage of different modes are smaller than the planned mileage. *L*_*j*_ is the planned mileage of the *i*th mode.

Model 2 (lower model): It describes the distribution of passenger traffic among different modes, essentially, the economic equilibrium in the market. The model is:

$$\text{max}\sum_{a\in A}\sum_{i=1}^{n}N_{a}^{i}\sum_{j=1}^{m}(V_{a}^{i}-C_{a}^{ij})\delta_{a}^{ij}$$ (2)

$$s.t.\left\{\begin{array}{l} \begin{array}{lll} (V_{a}^{i}-C_{a}^{ij})\delta_{a}^{ij}\geq 0, & \forall i=1,2,\ldots ,n;\;\forall j=1,2,\ldots ,m;a\in A & \left(h\right)\\ p_{a}^{j}-(\frac{F_{a}^{j}}{\sum_{i=1}^{n}N_{a}^{i}\delta_{a}^{ij}}+AV_{a}^{j})\geq R_{a}^{j}, & \forall i=1,2,\ldots ,n;\;\forall j=1,2,\ldots ,m;a\in A & \left(i\right)\\ \sum_{j=1}^{m}\delta_{a}^{ij}\leq 1, & \forall i=1,2,\ldots ,n;\;a\in A & \left(j\right)\\ 0\leq \;\delta_{a}^{ij}\leq 1, & \forall i=1,2,\ldots ,n;\;\forall j=1,2,\ldots ,m;a\in A & \left(k\right) \end{array}\\ \begin{array}{l} C_{a}^{ij}=P_{a}^{j}+\beta_{a}^{i}t_{a}^{j}+\gamma_{a}^{i}t_{a}^{j},\\ F_{a}^{j}(k+1)=F_{0}{}_{a}^{j}+\sum_{r}h_{a}^{jr}X_{a}^{jr}(k)L_{doc}{}_{a}^{jr}+\sum_{r}g_{a}^{jr}Y_{a}^{jr}(k)L_{doc}{}_{a}^{jr} \end{array} \end{array}\right.$$

The objective function (2) represents the pursuit of maximum consumer surplus in the whole society under the condition of relatively high personal travel surplus. It indicates that the fundamental goal of transport corridor supply is to meet the demand to the maximum extent. Constraint (h) shows that the demand entity *i* choose the *j*th mode on the basis that the trip surplus is greater than 0; otherwise, the trip will be meaningless. From an economic perspective, constraint (i) shows that on a specific transport corridor, if the turnover of the *j*th mode at *P*_*j*_ price is no less than *Q*_*j*_, the mode will have sustainable market vitality on the corridor and vice versa.

### A property of the optimal solutions

For the above passenger corridor mode configuration problem,we observe a property for the optimal solutions, and the details are as follows.

#### Property 1

If any travel demand subject *i* can choose a transportation mode that can maximize the residual value of its own travel (at least acceptable), and if any transportation mode can realize its own interest requirements, and at the same time the social benefits of the entire transportation system are better, then it is optimal to configure the ability of various modes of transport in the corridor.

#### Proof

In order to fully reflect the heterogeneity of demand and avoid the fatal flaw of utility theory in the measurement of travel utility, let *D*_*i*_ denote the consumer surplus value of travel demand entity, then it can be calculated as *D*_*ij*_ = *V*_*i*_-*C*_*ij*_. The transport demand subject *i* always hopes to choose the transport mode *j* that can achieve the largest consumer surplus, but when the supply of the *jth* transport mode is insufficient, it can only choose other transport modes *k* with the second highest consumer surplus (*k*≠ *j*), if the travel surplus is negative, the demand subject *i* will give up the trip, that is, the decision criterion for the demand subject to choose the travel mode can be expressed as *max*(*V*_*i*_*-C*_*ij*_). At this time, the consumer surplus of the entire transportation corridor is $\text{max}\sum_{a\in A}\sum_{i=1}^{n}N_{a}^{i}\sum_{j=1}^{m}(V_{a}^{i}-C_{a}^{ij})\delta_{a}^{ij}$.

For the supply subject of the transportation market, that is, the *j*th transportation mode in the OD pair, when the demand intensity of a certain transportation mode *j* is not enough to make the transportation mode *j* realize the basic equilibrium return during the planning period, it will temporarily not be able to meet this demand. On the contrary, if the transportation volume *q*_*j*_ that the transportation mode *j* may complete can make it obtain the basic equilibrium reward, then this transportation mode has sustainable market vitality in this corridor. Therefore, we think whether a certain transportation mode to be able to provide transportation services, the following conditions should be met: $p_{a}^{j}-(F_{a}^{j}/q_{a}^{j}+AV_{a}^{j})-R_{a}^{j}\geq 0$

In addition, for corridor planners, the construction cost, environment and energy consumption are comprehensively considered on the basis of the above-mentioned equilibrium of supply and demand, so as to finally determine the capacity allocation of corridor transportation modes.

If the requirements of any of the above stakeholders are not met, the model will not be able to obtain the optimal solution.

### Model solving algorithm

The decision-making process proposed in this paper is a system optimization problem with two-level hierarchical structure, which is essentially a nonconvex bi-level programming model.

When solving the lower-level model, we use the built-in Global search algorithm of MATLAB as the carrier, use the fmincon solver with the interior point algorithm as the kernel to establish the optimization object, and replace the algorithm kernel of the solver when the scale of the calculation example changes. Since the upper model is a mixed integer linear programming problem (MILP), the intlinprog algorithm is used to solve it. The specific steps are as follows.

Step 1: Parameter initialization. Give the initial travel intention **δ**(0), fare rate **p**(0) and line information. Set related cycle flag to zero (for example, the current cycle number *k* = 0).
Step 2: Solve the lower model. Substitute the initial values of **δ**(0) and fare rate **p**(0) into the lower model for **δ**(*k*) and **p**(*k*).
Step 3: Solve the upper model. Obtain the passenger flow of each mode **q**(*k*) according to travel wishes **δ**(*k*) and the number of travel entities *N*. Then substitute it into the upper model for **X**(*k*) and **Y**(*k*) and update relevant parameters.
Step 4: Cycle calculation. Solve the lower model with the new **X**(*k*) and **Y**(*k*) for **δ**(*k*+1) and **p**(*k*+1). Then solve the upper model for **X**(*k*+1) and **Y**(*k*+1).
Step 5: Convergence judgment. If |*Z* (*k* + 1) − *Z*(*k*)| ≤ *ε*, stop; Otherwise, let *k* = *k*+1, and go back to step 2. And *ε* is the iterative accuracy.

**Fig 1:**
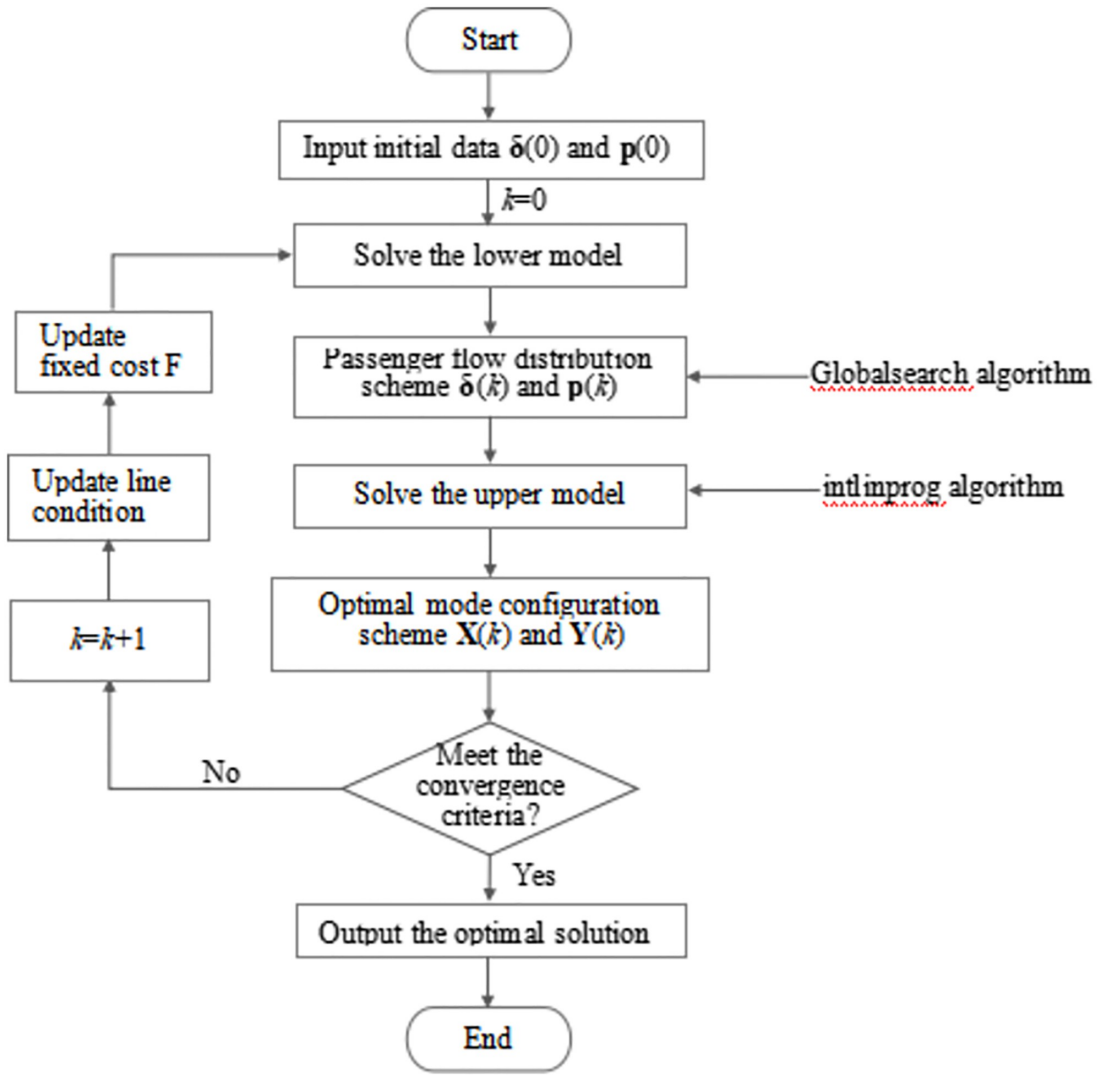
Flow chart of optimization solution.

The flow chart of the model optimization algorithm is shown in Fig 1.

## Computational experiments

This section takes Beijing-Shanghai Route (representative of medium and long-distance passenger transport market) and Shanghai-Nanjing Route (representative of short-distance passenger transport market) in Beijing-Shanghai transport corridor as examples to verify the effectiveness of the model and algorithms. First, the detailed information of the corridor is summarized, and then the influence of parameter changes on the results is analyzed. Finally, some management suggestions are proposed, which can be used for corridor layout planning.

### Case data

Beijing-Shanghai corridor is one of the eight verticals of China’s “Eight Verticals and Eight Horizontals” railway project. It is one of the busiest transportation corridors in China, and runs through Beijing, Tianjin, Hebei, Shandong, Jiangsu, Shanghai and other provinces and cities, connecting the Bohai area and Yangtze River Delta metropolitan areas. By referring to the relevant data [30] of “Feasibility Report of New Beijing-Shanghai High-speed Railway” of the Third Railway Survey and Design Institute, some data are estimated. Table 2 lists the current characteristics of various modes of transportation in Beijing-Shanghai transport corridor. Highway is not listed due to its extremely low market share in the medium and long- distance passenger transport market. Besides, in the short-distance transport market, civil aviation routes are generally not available.

**Table 2:** Parameter settings for the Beijing-Shanghai corridor.

Attributes	Beijing-Shanghai (OD1)			Shanghai-Nanjing (OD2)		
	Rail	High-speed railway (HSR)	Air	Rail	High-speed railway (HSR)	Highway
**Sharing fixed cost *F* (10,000¥/year km)**	212.47	1,415.1	2	235.85	1,415.1	85
**Unit variation cost *AV* (¥/person km)**	0.025	0.107364	0.52	0.025	0.107364	0.068
**Travel time *t* (h)**	16.9	9	4.95	5.11	4	4.11
**Distance (km)**	1,463	1,318	1,178	307	295	274
***Φ* (¥/kwh)**	0.7	0.7	5,800	0.7	0.7	5,800
***E* (kwh/person km)**	0.0021	0.0063	0.0000381	0.0021	0.0063	0.00000923
***E* (MJ/person km)**	0.00756	0.02268	1.270318	0.00756	0.02268	0.1864

Relevant data about the transportation demand of Beijing-Shanghai corridor is shown in Table 3, and we use the hypothetical demand data generated by sample surveys. This paper is only used for example analysis, and its main purpose is to clarify the theory and model methods generally. Therefore, the values of various parameters do not affect the application value of the results, as long as they are assigned reasonably in practical applications. Referring to the study of Wang et al (2021) [6], this paper divides the main travel demand into consumption, production and emergency travel.

**Table 3:** Main parameter assumptions for certain travel demand entities.

Attributes	Consumer travel			Productive travel		Emergency travel
	Group 1	Group 2	Group 3	Group 4	Group 5	Group 6
**Distribution *A***	10%	15%	10%	30%	30%	5%
**Distribution *B***	30%	10%	10%	30%	15%	5%
**Travel value *V* (¥/trip)**	2,000	4,000	8,000	5,000	10,000	20,000
**Time value coefficient *β* (¥/hour)**	12.5	30	60	40	80	500
**Other psychological cost coefficient (¥/hour)**	5	10	10	10	20	20

It should be noted that the fixed cost in Table 2 is estimated by moderate adjustment on the basis of the construction cost. To determine *F*, reference can be found in Tzeng’s research [31]. The treatment costs of CO, NO_2_ and C_x_H_y_ are 92.7 yuan/kg, 3100 yuan/kg and 7750 yuan/kg, respectively, among which the treatment costs of NO_x_ are approximately equal to that of NO_2_. The economic conversion coefficient *φ* of energy consumption is calculated directly with reference to energy price. Beijing, Nanjing and Shanghai, three major cities along the Beijing-Shanghai Corridor, have an upper limit of bearing capacity of environmental pollution caused by urban external passenger transport of 6.04*10^8g/day, 2.28*10^8g/day and 6.87*10^8g/day [32], respectively. And their upper limits of bearing capacity of energy consumption are 7.39*10^7MJ/day, 2.79*10^7MJ/day and 8.41*10^7MJ/day [32], respectively. The upper limit of each OD for different transportation modes is determined by the upper limit of environmental pollution bearing capacity and the upper limit of total energy consumption.

### Solution and discussion

There are vast differences in passenger travel demands in different distance transport corridors, so the corridor passenger transport markets are definitely diversified. This section analyzes the optimization of transportation modes of Beijing-Shanghai transport corridor (OD1) and Shanghai-Nanjing transport corridor (OD2), to observe the differences of mode configuration of corridors with different distances. All numerical experiments are performed on MATLAB 2019a. In the model, the lower-level is solved with fmincon, and the upper-level is solved with intlinprog function.

It is assumed that the transportation demands of Beijing-Shanghai section and Shanghai-Nanjing section are both 80 million trips. The basic profit rate of high-speed rail is 3%, the basic profit rate of other modes is 4%, and all kinds of travel demand entities are distributed as a combination A (hereafter referred to as the Base-Case). The results are solved by the above model and algorithms. Table 4 presents the results for the Base-Case solution, obtained after 38341.66s of CPU time. They show that under the given parameters, the Beijing-Shanghai section needs to be equipped with three modes: general railway, high-speed railway, and civil aviation. Among them, the general railway bears 2.4% of the total transportation demand. As the passenger flow is relatively inadequate, the equilibrium fare of the general railway is basically stable around 1,704.25 yuan to achieve breakeven operation. Equilibrium fare refers to the price established in the process of supply and demand interaction to complete its passenger transportation. It ensures the obtaining of the basic profit rate of return and has some reference value in an actual planning process. High-Speed rail bears 92.6% of the passenger flow, which is mainly concentrated in the second and third categories of consumer travel and production travel, and the equilibrium price is around 532.29 yuan. It is found that the transportation capacity of the existing high-speed rail cannot meet the demand, and a new high-speed rail is in urgent need. The civil aviation only bears 5% of the corridor demand, which is characterized by emergency travel. It aims at “fast travel” and has nothing to do with the identity of travelers. In such situation, the equilibrium fare of civil aviation is 665.57 yuan.

**Table 4:** Base-case solution.

OD	Mode	Market share (%)	Equilibrium price (¥)	X	Y
**Beijing-Shanghai (OD1)**	Rail	2.4	1,704.25	0	0
	I	--	--	0	0
	II	--	--	0	0
	HSR	92.6	532.29	1	0
	Air	5	665.57	0	0
**Shanghai-Nanjing (OD2)**	Rail	0.48	1,910.58	0	0
	I	--	--	0	0
	II	--	--	0	0
	HSR	35	189.61	0	0
	Highway	64.52	38.64	1	0

Note: 1 for yes, 0 for no.

According to Table 4, there are only two modes to be configured in the Shanghai-Nanjing section: high-speed rail and expressway. Among them, expressway bears a larger proportion of the transportation volume, reaching 64.52%, and the corresponding balanced fare is only 38.64 yuan. Because there are plenty of demand entities who would choose expressway, the existing expressway capacity is overloaded and can hardly meet the demands. To achieve a balance between the supply capacity and demand, it is necessary to build a new expressway within reasonable investment under the constraints of environment and energy consumption. The high-speed rail bears 35% of the passenger demand in this section, and the balanced fare is 189.61 yuan while the general railway only bears 0.48% of the passenger traffic, which is negligible. For the Base-case, most of demand entities give priority to speed and time, and do not care much about fares. Therefore, few of them are willing to choose the general railway, which makes it difficult to obtain the basic equilibrium rate of return and realize the smooth value flow of the general railway system.

The two-level programming model proposed in this paper comprehensively considers the transportation demand characteristics, supply characteristics and constraints of the external environment, energy consumption and other parameters. It also considers the relationship between the existing transportation network transformation and the new network layout, which better reflects the actual situation. In order to verify the effectiveness of the model and algorithm, please see the next part of the content.

### Parameter impact analysis

Demand can change in its level, its distribution or both. Also, on the supply side, the scale and type of service in corridor should be modified accordingly [33]. In this part, we will discuss how corridor demand and demand distribution affect transportation modes. In addition, we also try to analyze the influence of equilibrium rate of return on the result of transportation modes.

#### Influence of corridor demand and distribution

**Fig 2:**
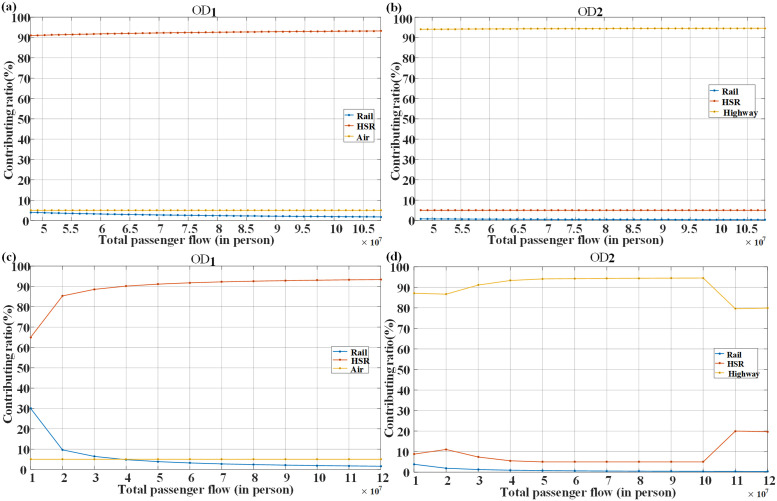
Influence of total corridor demand on market share (a) OD1 (Distribution A); (b) OD2 (Distribution A); (c) OD1 (Distribution B); (d) OD2 (Distribution B).

**Fig 3:**
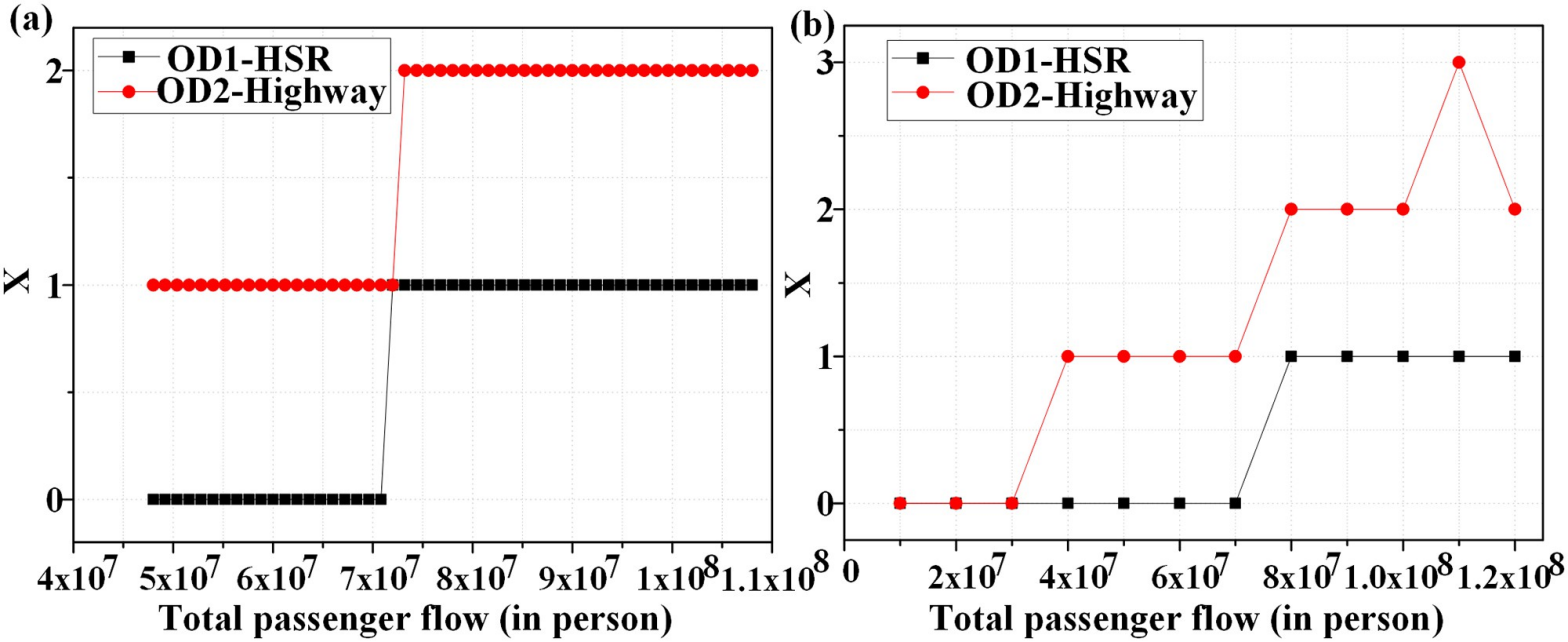
Influence of demand on corridor configuration (a) Distribution A; (b) Distribution B.

So far, the previous results have been obtained under established parameter assumptions. Obviously, in different stages of economic development, the transportation demand of a corridor will also change greatly. For this reason, we repeat the computations under the condition of increasing demand for transport corridor. Fig 2 shows the change in the share of transport modes that is affected by the demand of certain corridor. As expected, the larger the transport demand in corridor, the larger the share of high-speed railway for medium and long-distance corridors, where the share of railway has fallen. We find that the market share of air is also constant in this experiment. The new-built corridor modes are shown in Fig 3. For OD1, when the corridor demand is less than 70.8 million, the existing transportation capacity can basically meet the demand, and no additional construction is needed. When the demand for the corridor continues to increase to 72 million, the passenger traffic shared by the high-speed rail is about 66.47 million, which is beyond the existing capacity of the high-speed rail. Therefore, it is necessary to build a new high-speed rail with the same distance. At this time, the balanced fare of the high-speed rail also rises to 586 yuan, which is due to the increase in construction cost caused by the new high-speed rail. In order to reach the basic balanced rate of return, it can only be compensated by increasing the passenger traffic and raising the fare. With the continuous increase in the demand for corridors, it is necessary to build a new high-speed railway. When the corridor demand distribution is combination B, and the corridor demand increases from 10 million to 120 million, the market share of high-speed rail increases from 65% to 93.39%, while that of general railway decreases from 30% to about 2%. If the corridor demand reaches 80 million or above now, a new high-speed rail should be built for the corridor. Compared with Combination A, the passenger traffic required for new high-speed rail is higher due to the large proportion of groups with low travel demand in Combination B. Thus, in order to open a new high-speed railway and reach the basic rate of return, it demands a higher passenger flow.

For OD2, we take Combination A as an example. It is found that with increased travel demand of the corridor, nearly 95% of the demand entities are willing to choose expressway, and a small number of passengers opt for the high-speed rail. When the demand of the corridor is between 48 million and 72 million trips, a new expressway needs to be built in the corridor. At this point, the equilibrium fare of the expressway also drops from 39.92 yuan (corresponding to 48 million) to 36.45 yuan (corresponding to 72 million). As the lower cost of new expressway construction is mainly offset by the rapidly increasing passenger flow of expressway, the basic equilibrium rate of return is realized, and the equilibrium fare of expressway decrease generally. When the demand for corridor transportation reaches 73.2 million trips or above, two new expressways need to be constructed. At this time, the overall cost of new construction is higher, and the balanced fare increases to 39.72 yuan (the fare is 36.45 yuan for 72 million trips) (see Fig 3). When the corridor demand distribution is Combination B, and the corridor demand is not higher than 100 million trips, the transportation mode is similar to that of Combination A. When the demand is between 100 million and 110 million, the passenger flow of expressway drops to 79.59%. On the contrary, high-speed railway is gradually favored by the demand entities. When the corridor demand reaches 110 million trips, three new expressways need to be built, and the equilibrium fare of expressway increases from 37 yuan to 40 yuan. At the same time, the equilibrium fare of high-speed rail has dropped dramatically, from 878.38 yuan to 230.27 yuan, and some travel demand entities prefer to high-speed rail among other options. This shows that corridor demand and demand distribution have a significant impact on the transportation mode.

#### Influence of the change of high-speed rail equilibrium return rate

**Fig 4:**
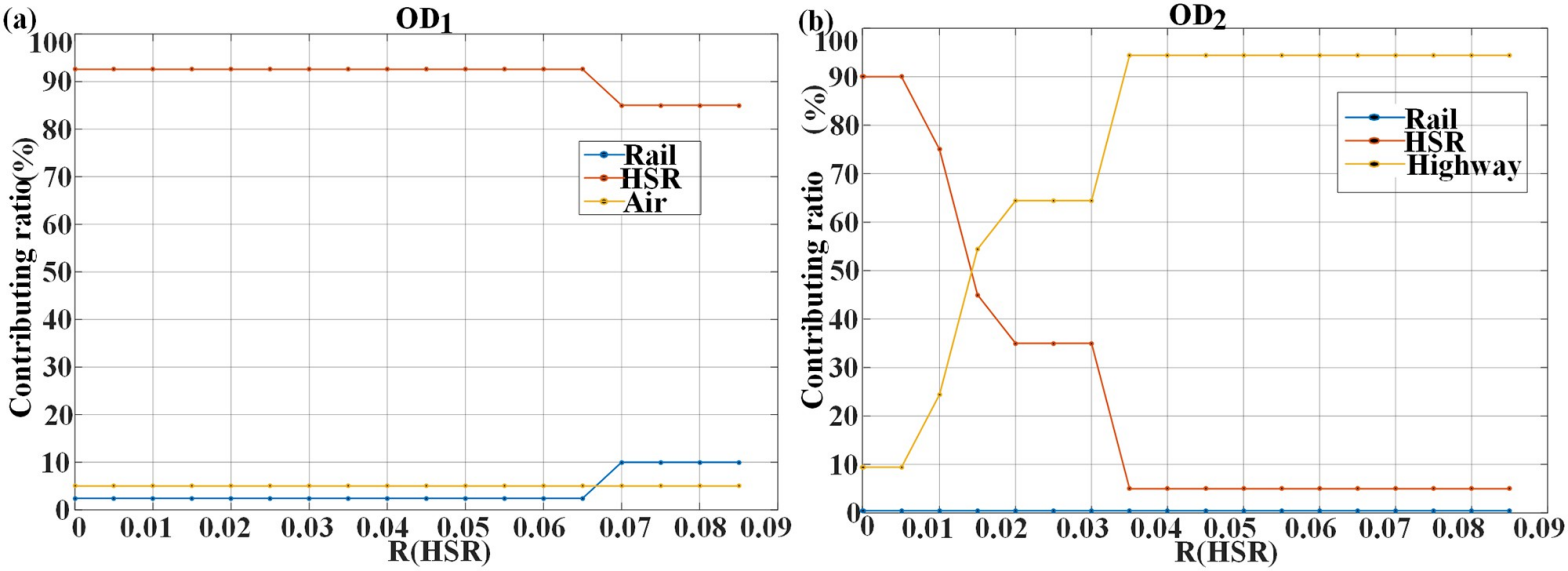
Impact of high-speed rail equilibrium return rate changes on mode structure (a) OD1; (b) OD2.

In order to explore the changes of transportation mode with the equilibrium return rate of high-speed rail, this paper analyzes the change of passenger flow sharing rate of various modes of transportation. The total demand is 80 million trips, the demand distribution is type A, and other parameters are shown in Table 3. The equilibrium return rate of various modes except high-speed rail is 4%, and the equilibrium return rate of high-speed rail is 8.5% from the pursuit of capital preservation operation (Fig 4).

The results show that for OD1 (Fig 4a), when R_HSR_ is lower than 6.5%, the transportation mode basically remains unchanged for general railway (2.4% of share rate), high-speed railway (92.6%) and civil aviation (5%), which is related to less price-sensitive travel demand during scenario simulation. When R_HSR_ is greater than 6.5%, high-speed rail requires for higher transportation demand and passenger fares in order to ensure its profit. For example, when the high-speed rail balanced return rate is 7%, the corridor transportation undertaken by high-speed rail drops to 85%, and the balanced fare is 616.35 yuan, 38 yuan higher than 578.41 yuan when the rate is 6.5%. Then some of the passenger flow is transferred to general rail, which accounts for nearly 10% of the corridor transportation. Due to sufficient passenger flow, the balanced fare of general rail drops to 483.65 yuan, and in this process, the demand for civil aviation transportation remains unchanged at 5%.

In Fig 4(b), it is found that with the increase of R_HSR_ in short-distance OD2 section, there is great difference in the capacity configuration of various transportation modes in the corridor. When R_HSR_ is 0.5% or below, only two modes are needed: high-speed rail and expressway. And the high-speed rail bears 90% of the transportation demand of the corridor with a equilibrium fare of 114 yuan. Under this condition, the existing high-speed rail transportation capacity is insufficient and it is overloaded, so it is imperative to build a new high-speed rail. On the other hand, the expressway only needs to be equipped with the transportation capacity that can meet 10% of the transportation demand, and the balanced fare is 60.44 yuan. When R_HSR_ reaches 2% to 3%, the market share of high-speed rail drops rapidly from 90% to 35% because high-speed rail needs a certain amount of passenger traffic to achieve its own economic sustainability, and the equilibrium fare rises from 114 yuan to 186.67 yuan. However, the market share of expressway gradually rises from 10% to 64.43%, and the equilibrium fare drops to 38.64 yuan. Subsequently, as the balanced return rate of high-speed rail continues to increase to 3.5%, the market share of high-speed rail drops to 5% again. In order to make its operation sustainable economically, the passenger fare required by high-speed rail reaches 1,085.63 yuan, and the market share of expressway is improved considerably, completing nearly 95% of the transportation demand. As the equilibrium rate of return continues to improve, the transportation capacity of high-speed rail remains almost unchanged, but its passenger fares gradually increase.

On the whole, the simulation results of the above scenarios basically conform to the economic operation mechanism, which shows that the model has goodfeasibility.

**Fig 5:**
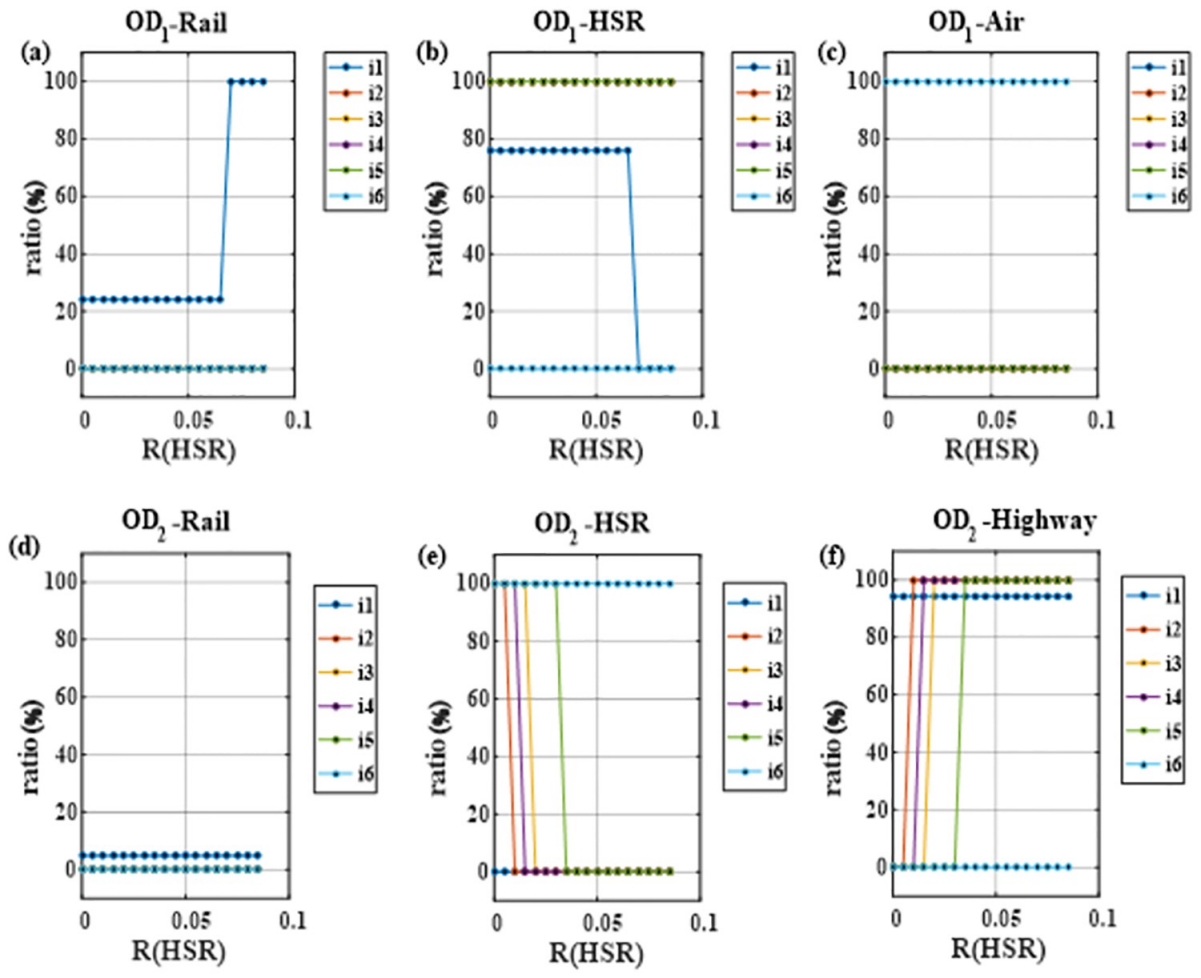
Influence of high-speed rail equilibrium return rate on entities’ willingness to choose various modes. (a) Passenger flow of general railway in OD1 section; (b) Passenger flow of high-speed railway in OD1 section; (c) Passenger flow of civil aviation in OD1 section; (d) Passenger flow of general railway in OD2 section; (e) Passenger flow of high-speed railway in OD2 section; (f) Passenger flow of highway in section OD2.

In order to clarify the choices of travel demand entities, Fig 5 is created. For OD1, when R_HSR_ is 6.5% or less, only 24.14% of the first type of demand entities will choose general railway, and the remaining 75.86% of the first type and the second to fifth types tend to choose high-speed rail due to economic consideration. And the sixth type of demand entities who travel for emergency such as medical care are more likely to choose flight. When the balanced rate of return of high-speed rail reaches 7% or above, the first type is willing to choose general railway, while the other remain unchanged. For OD2 shown in Fig 5(d)–5(f), when the equilibrium return rate of high-speed rail is 0.5% or less, the second to sixth types of demand entities tend to choose high-speed rail, and most of the first type are willing prefer expressway. When the equilibrium return rate of high-speed rail increases to 1%, the second type gives up high-speed rail for expressway, and the choice of other groups does not change. When the equilibrium return rate of high-speed rail continues to increase to 1.5%, it is difficult for the fourth group to accept the high fare of 152 yuan for high-speed rail, and then they opt for the expressway. When the equilibrium return rate of high-speed rail increases to 2%, the third type gives up high-speed rail travel and chooses expressway. At this point, the equilibrium fare of high-speed rail is 186.67 yuan, while the fare of expressway is only 38.64 yuan. When the equilibrium rate of return of high-speed rail continues to increase to 3.5%, the equilibrium fare of high-speed rail increases sharply, reaching 1085.6 yuan, and the fifth group chooses expressway in order to maximize their travel surplus under the high fares. Under such circumstances, the market share of expressway reaches 94.44%, and the existing expressway transportation capacity is no longer adequate to match the demand, so it is urgently necessary to build two new expressways to achieve balance between supply and demand. This shows that as the equilibrium rate of return of a transportation mode becomes higher, the corresponding market share will show a downward trend. Consequently, the equilibrium price will rise.

Table 5 reflects the influence of the change of high-speed rail equilibrium return rate on the new construction of transportation mode. For OD1, when the high-speed rail equilibrium return rate is less than 7%, the existing high-speed rail transportation capacity is insufficient and a new high-speed rail needs to be built. When the equilibrium rate of return of high-speed rail exceeds 7%, the demand for corridor transportation undertaken by high-speed rail decreases to 68 million trips, so the transportation capacity of existing high-speed rail is sufficient and no new construction is needed. For OD2, when the equilibrium return rate of high-speed rail is 0.5% or less, a new high-speed rail needs to be built. When the equilibrium return rate of high-speed rail is between 1.5% and 3%, a new expressway needs to be built because its market share is increasing gradually. When the equilibrium return rate of high-speed rail reaches 3.5% or above, two new expressways need to be built, so as to realize the optimal allocation of transportation network resources in the corridor and meet the transportation demand to the maximum extent.

**Table 5:** New construction of various transportation modes in the corridor.

R(HSR)	OD1			OD2		
	Rail	HSR	Air	Rail	HSR	Highway
**0**	0	1	0	0	1	0
**0.005**	0	1	0	0	1	0
**0.01**	0	1	0	0	0	0
**0.015**	0	1	0	0	0	1
**0.02**	0	1	0	0	0	1
**0.025**	0	1	0	0	0	1
**0.03**	0	1	0	0	0	1
**0.035**	0	1	0	0	0	2
**0.04**	0	1	0	0	0	2
**0.045**	0	1	0	0	0	2
**0.05**	0	1	0	0	0	2
**0.055**	0	1	0	0	0	2
**0.06**	0	1	0	0	0	2
**0.065**	0	1	0	0	0	2
**0.07**	0	1	0	0	0	2
**0.075**	0	0	0	0	0	2
**0.08**	0	0	0	0	0	2
**0.085**	0	0	0	0	0	2

### Managerial insights

It is suggested that the government departments should carefully analyze the transportation demands, understand the overall structure, and estimate the parameter data of various transportation modes in configuration. They also should comprehensively consider the construction periods, future industrial structure, economic development level, population size and structure, urban development status and other factors. Rational planning should be formualted according to the theoretical basis and the model and algorithms proposed in this paper, so as to improve the utilization of scarce resources and minimize resource waste.

In addition, we can compare the theoretical comprehensive transportation corridor mode structure obtained in this paper with the actual mode structure, seek the deviation of the realistic corridor mode structure, and then provide corresponding solutions through local engineering technical and economic analysis. The current research can provide systematic theoretical support for the comprehensive transportation network planning and transportation hub planning in the future. Meanwhile, the economic equilibrium theory and methods used in this paper can provide support for various transportation modes such as road and railway to seek reasonable market positioning, optimize transportation organization, and achieve optimal utilization of stocks.

## Conclusion

The contribution of this paper lies in its theoretical model, algorithm solutions and case studies for corridor structure optimization from the perspective of different entities. Through literature review and analysis, it is found that the existing researches focus mainly on the balance of quantity, but have not considered the internal mechanism of transportation supply in relation to demand, that is, the economic balance between supply and demand. The interest demands of different participants in corridor planning are not considered.

1. In this paper, a bi-level programming model of corridor mode structure optimization is constructed, which considers the corridor planner, the supply entity and demand entity. This model realizes an economic balance between supply and demand, and changes the traditional situation of quantity balance. In addition, it considers new network layout, existing transportation network transformation and technical issues of the rail.
2. For the bi-level programming model, this paper uses the algorithms of intlinprog and Globalsearch to solve practical problems. It also shows how to determine the optimal solution of economic equilibrium between supply and demand and the optimal solution of some parameter changes.
3. The research shows that the capacity allocation of different transportation modes is quite different in corridors with different distances. The advantages of high-speed rail in medium and long-distance transportation corridors are obvious. In addition, the total amount and composition of transportation flow and the equilibrium return rate have significant impact on the mode allocation, and the influence of parameter changes accords with the economic mechanism. This indicates that the model and algorithms are effective, providing auxiliary decision support for the coordinated development of various transportation modes in the corridor and the orderly construction of infrastructure.

However, our model focuses mainly on the structural optimization of parallel transportation corridors with a single OD. In fact, the transportation corridor structure may be complicated. We need to simplify the complex transportation corridor to a single OD and then integrate it. In addition, the model will be expanded in the future to adapt to uncertain corridor requirements, and sensitivity analysis of parameters will be introduced. The research ideas in this paper can serve as reference for the configuration of multi-OD corridors, regional comprehensive transportation network planning, urban comprehensive transportation network planning, market positioning of a certain transportation mode, transportation organization optimization and other issues.
